# Differing hypertrophy patterns from open and closed kinetic chain training affect quadriceps femoris center of mass and moment of inertia

**DOI:** 10.3389/fphys.2023.1074705

**Published:** 2023-03-14

**Authors:** Jacob E. Earp, Domenic Angelino, Disa L. Hatfield, Vincent Colantuono, Euan R. Jackson, Kristin D. Morgan, Alessandra Adami, Kathleen J. Melanson, Anthony J. Blazevich

**Affiliations:** ^1^ Department of Kinesiology, University of Connecticut, Storrs, CT, United States; ^2^ Department of Kinesiology, University of Rhode Island, Kingston, RI, United States; ^3^ Department of Biomedical Engineering, University of Connecticut, Storrs, CT, United States; ^4^ Department of Nutrition, University of Rhode Island, Kingston, RI, United States; ^5^ Centre for Exercise and Sports Science Research (CESSR), School of Medical and Health Sciences, Edith Cowan University, Joondalup, WA, Australia

**Keywords:** regional hypertrophy, inhomogeneous hypertrophy, center of mass, moment of inertia, biomechanics, muscle shape, running economy, mass distribution

## Abstract

**Purpose:** To determine whether kinetic chain pattern during knee extensor strength training influences quadriceps femoris center of mass and moment of inertia about the hip in a predictable manner as such changes can affect running economy.

**Methods:** Twelve participants completed 8 weeks of both unilateral open (OKC) and closed (CKC) kinetic chain resistance training on opposing legs. Changes in quadriceps femoris muscle volume (VOL_QF_), center of mass location (CoM_QF_), and moment of inertia (*I*_QF_) about the hip were determined from magnetic resonance images scans. Regional hemodynamics of the vastus lateralis taken at 30% and 70% of muscle length during OKC and CKC bouts early in the training program were measured using near-infrared spectroscopy (NIRS) and used *post hoc* to predict changes in CoM_QF_.

**Results:** While increases in VOL_QF_ were similar between OKC (Δ79.5 ± 87.9 cm^3^) and CKC (Δ60.2 ± 110.5 cm^3^, *p* = 0.29), the patterns of hypertrophy differed; a distal shift in CoM_QF_ (Δ2.4 ± 0.4 cm, *p* < 0.001) and increase in *I*_QF_ (Δ0.017 ± 0.014 kg m^2^, *p* < 0.001) occurred in OKC but not in CKC (CoM_QF_: Δ-2.2 ± 2.0 cm, *I*_QF_: Δ-0.022 ± 0.020 kg m^2^, *p* > 0.05). Regional hemodynamics assessed by NIRS during a single training session displayed similar exercise and regional differences and predicted 39.6% of observed changes in CoM_QF_.

**Conclusions:** Exercise selection influences muscle shape sufficiently to affect CoM_QF_ and *I*_QF_, and these changes may be predicted in part from NIRS measurements during a single workout. Given *I*_QF_ is inversely related to running economy and since CKC exercise provides a more proximal pattern of hypertrophy than OKC, it may be more preferential for running. The results from the present study also highlight the potential of NIRS as a tool for predicting patterns of hypertrophy between different exercises and exercise conditions.

## Introduction

A skeletal muscle’s physical structure is the primary determinant of its functional capacity (Burkholder et al., 1994; Blazevich et al., 2006; Blazevich et al., 2009). In the quadriceps femoris (QF), variations in muscle cross-sectional area, fascicle length, and fascicle angle have been observed both between the muscular components and along the lengths of each component (Blazevich et al., 2006). Such structural variations allow for different regions within a single muscle to be more specialized for certain exercise conditions than others (Blazevich et al., 2006). Previous studies have also reported intramuscular (i.e., proximo-distal) variations in muscle activity, oxygenation, stress and strain between exercises that vary by movement pattern, force level, and movement speed (Shin et al., 2009; Wakahara et al., 2012; Miyamoto et al., 2013; Wakahara et al., 2013; Earp et al., 2015; Wakahara et al., 2017). Consistent with this, differing intramuscular hypertrophic patterns have been reported after training lasting at least several weeks with exercises using different movement patterns (Wakahara et al., 2012; Miyamoto et al., 2013; Nunes et al., 2020), loads and speeds (Earp et al., 2015) and contraction modes (concentric vs eccentric) (Franchi et al., 2014). Thus, regional hypertrophic variation may not only result from genotypic influence but also from physical loading and unloading patterns over time.

The possibility of using specific exercises to emphasize regional adaptations has important applications in both athletic and clinical populations. For instance, clinical exercise programs could be tailored to target portions of a muscle that are subject to injury (Askling et al., 2008) or known to influence joint stability (Castanov et al., 2019). Hypertrophic patterns in response to loading may also directly affect limb mechanics (Handsfield et al., 2017) and influence muscle strength expression after training (Noorkõiv et al., 2014; Trezise et al., 2016; Trezise and Blazevich, 2019) 2/7/23 3:47:00 PM. For instance, while running, an increase in muscle mass that results from resistance training will also increase the limb’s inertial resistance and can potentially reduce running performance and economy (Myers and Steudel, 1985; Hale, 1990; Handsfield et al., 2017). Thus, training that preferentially stimulates proximal hypertrophy may minimize increments in moment of inertia and theoretically optimize movement performance in tasks requiring high movement speeds or endurance. This is important when considering that the moment of inertia of the thigh and shank about the hip is the primary resistance to motion during the recovery (swing) phase in running. In this case, hypertrophy without significant distal, or even with proximal, shift in center of mass location would be preferential as it minimizes the total change in inertia as mass is added (Myers and Steudel, 1985). Consistent with this, faster runners possess greater proximal and less distal muscle mass than slower runners at both the 100 m and marathon distances (Abe et al., 2000; Kumagai et al., 2000) and compared to non-sprinters (Handsfield et al., 2017), despite unequivocal results relating QF volume to running performance (Sugisaki et al., 2011; Handsfield et al., 2017; Sugisaki et al., 2018; Miller et al., 2021).

Little is known about the effects of exercise movement pattern or other programming variables on region-specific hypertrophy in response to prolonged resistance or other exercise training. In QF, for example, concentric-only knee extension strength training has been shown to trigger more proximal hypertrophy than eccentric-only training (Franchi et al., 2014) whilst high-load, slow-velocity squat lift training resulted in more proximal hypertrophy than light-load, fast-velocity jump squat training (Earp et al., 2015). However, such exercise manipulation is not always feasible as training programs should normally contain both eccentric and concentric contractions and include exercises performed across the load-velocity spectrum (Haff et al., 2016). Nonetheless, kinetic chain pattern may be exploitable since open kinetic chain (OKC) knee extension exercises produce very different muscle force and activity patterns than closed kinetic chain (CKC) squat and leg press exercises (Escamilla et al., 1998). Additionally, previous studies have independently observed that OKC (knee extension) exercise results in a distal pattern of hypertrophy (Blazevich et al., 2007) whilst CKC (squat-lift) training results in a similar relative increase in CSA in the proximal and distal thigh after 8 weeks of training (Δ15.4% in both location) but a larger absolute increase in cross-sectional area in the larger proximal region (Δ12.2 cm^2^) than the smaller distal region (Δ9.4 cm^2^) (Earp et al., 2015). These differences in hypertrophy patterns may be attributed to differing movement kinetics or loading patterns between the exercises where external resistance increases during the concentric phase during a plate loaded knee extension and decreases during the concentric phase of a squat (Wallace et al., 2018). However, no studies have directly compared region-specific QF hypertrophy between OKC and CKC exercises under similar training conditions (relative external load, range of motion, movement speed, and training volume) despite their differential use in athletic and rehabilitation programs.

Given the previously observed changes regional hypertrophy in response to different training conditions and their potential impact these changes on running performance, there is a clear benefit to developing methods to predict hypertrophy patterns in response to different training stresses. Along these lines, previous studies have found that patterns of hypertrophy can be predicted from region-specific, post-exercise changes in intramuscular fluid accumulation measured using T_2_-weighted MRI (Wakahara et al., 2012; Wakahara et al., 2013; Wakahara et al., 2017), while regional muscle activity during exercise measured by electromyography (EMG) are unrelated to patterns of hypertrophy (Miyamoto et al., 2013; Earp et al., 2016). Presently, no studies have determined if patterns of hypertrophy can be predicted by near infrared spectroscopy (NIRS), which provides information about changes in localized muscle oxygenation and is more fundamentally similar to T_2_-weighted MRI than EMG while having a lower research cost compared to T_2_-weighted MRI.

As QF is the primary knee extensor (Blazevich et al., 2009) and highly responsive to strength training (Franchi et al., 2014; Earp et al., 2015), differing patterns of QF hypertrophy between OKC and CKC may substantially influence moment of inertia of the QF about the hip (*I*_QF_) and thus performance across a variety of movement tasks (Abe et al., 2000; Kumagai et al., 2000). The purpose of the present study, therefore, was twofold: (1) to determine whether exercise kinetic chain pattern (OKC vs CKC) significantly influences center of mass location and moment of inertia of the QF about the thigh after 8 weeks of strength training; (2) to determine whether patterns of regional hypertrophy can be predicted from regional muscle activity during a single exercise bout performed in each condition. To develop our hypotheses, we completed a pilot study in which regional muscle oxygenation was measured in ten resistance trained men during OKC and CKC exercise. In this experiment, we found similar tissue oxygenation changes in the proximal region but 2.3 fold greater distal oxygenation in OKC than CKC during a single set of exercise. Given these results, and the observation that regional differences in muscle oxygenation tend to align with regional hypertrophic variations (Miyamoto et al., 2013; Wakahara et al., 2017), we hypothesized that patterns of regional hypertrophy specific to each exercise condition could be predicted from regional muscle activity during a single exercise bout, and that CKC (squat lift) training should thus promote similar proximal but less distal hypertrophy than OKC (knee extension) training. These patterns of hypertrophy should then result in a more proximal center of mass location and smaller limb moment of inertia, which have the potential to improve running performance and economy.

## Materials and methods

### Participants

Seven men (age: 22.3 ± 4.4 years, height: 171.0 ± 7.6 cm, body mass: 66.5 ± 9.5 kg, % body fat: 14.2 ± 4.6) and five women (age: 19.8 ± 0.8 years, height: 163.8 ± 4.3 cm, body mass: 58.6 ± 6.3 kg, % body fat: 24.4% ± 3.1%) aged 18–35 years completed this study. Each participant was moderately physically active but non-habitually resistance trained, defined as averaging ≤1 resistance training sessions per week over the last 6 months. Participants were excluded from the study if they had any clinical contraindications to lower body exercise or had experienced any significant lower body muscle, joint, tendon or bone injury that may have affected data collection, participation in the resistance training program, or otherwise affected results. Before enrollment, participants completed both an exercise and health history questionnaire to ensure they met the inclusionary criteria mentioned above. These questionnaires were reviewed by an independent medical consultant to determine eligibility based on the outlined criteria when any previous lower body injury, medical condition, or prescribed medication was indicated. After providing informed written consent, participant height was measured using a stadiometer and body mass and body fat percentage were measured by an 8-polar, multifrequency bioelectrical impedance assessment (InBody 770, Seoul, Korea), after which they were familiarized to all research protocols and exercise techniques. During the study, participants were not allowed to engage in other lower body resistance training activities, were required to maintain their current physical activities outside of the study and could not make any substantive changes to their diet. The study was approved by the institutional review board at the University of Rhode Island.

### Experimental design

The present study utilized a within-participant design with repeated measures in which each participant simultaneously completed 8 weeks of progressive unilateral OKC (single-leg knee extension) and CKC (single-leg squat) training using contralateral limbs. The order of training within each training session (OKC before CKC or CKC before OKC) as well as the assignment of training condition to participants’ dominant and non-dominant limbs were randomized using a block randomization table in a counterbalanced manner. All imaging and analyzes were performed by researchers who were blinded to the experimental condition being examined.

At least 48 h after familiarization, a series of magnetic resonance images (MRI) were taken along the length of each thigh from which quadriceps femoris muscle volume (Vol_QF_) was measured and center of mass location (CoM_QF_) and thigh moment of inertia (*I*_QF_) were calculated (as described below). Within 7 days of the first MRI assessment, participants started an 8-week training intervention for both OKC and CKC on contralateral limbs. On the first and last days of training, participants completed a strength test for both OKC and CKC exercises on their assigned legs. During the second week, participants completed their first training session while wearing NIRS sensors over their proximal and distal vastus lateralis so that regional changes in oxygenated (HbO_2_) and deoxygenated (HHb) hemoglobin concentrations and relative oxygen saturation (StO_2_) in response to OKC and CKC could be compared. MRI imaging was then repeated 4–5 days after the last training session and thus 48–72 h after the second (post-training) strength test.

Comparisons were then made between conditions over time (interaction effect) for Vol_QF_, CoM_QF_ and *I*_QF_ as well as within each condition over time (training effect) for Vol_QF_, CoM_QF_, *I*_QF_, and maximum strength for both exercises. Acute changes in HbO_2_, HHb and StO_2_ during a single bout of OKC and CKC were compared between conditions and between regions (proximal and distal), and the interaction between condition and region was tested. The predictive strength of acute HbO_2_, HHb and StO_2_ responses on each individual’s changes in CoM_QF_ and *I*_QF_ after 8-week of training were then calculated.

### MRI scanning and analysis

**FIGURE 1:**
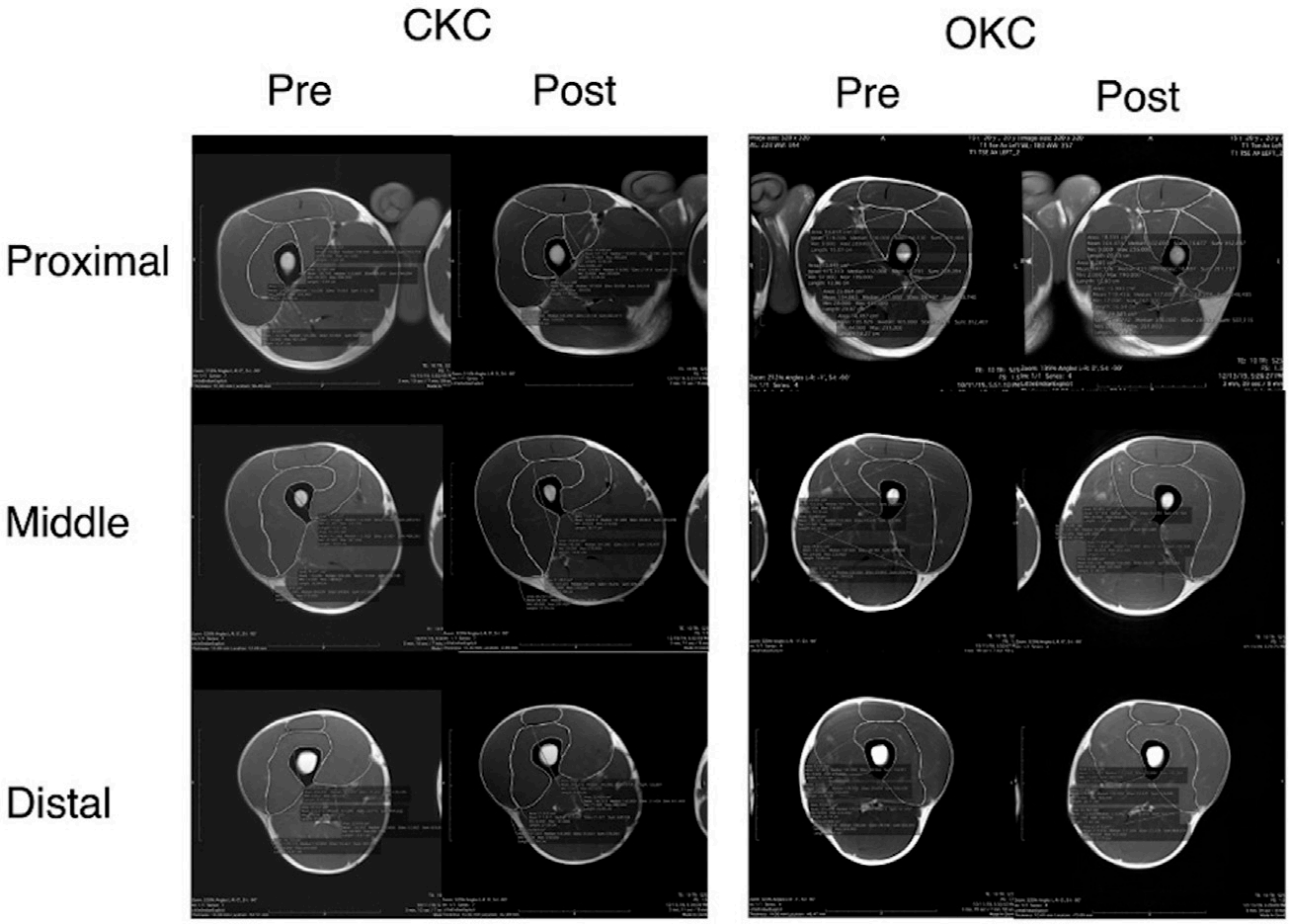
A representative subject MRI results. Figure reports the measurements of each component of the quadriceps femoris at 33% (Proximal), 50% (Middle) and 67% (Distal) of the distance from the femoral head to the tibial tuberosity. Images are before (Pre) and after (Post) 8 weeks of closed kinetic chain (CKC) or open kinetic chain (OKC) resistance training program.

Participants had MRI scans of their lower body taken before and after the training intervention. The former was obtained at least 48 h after any exercise or strenuous physical activity while the latter was performed between 96–120 h after the final training session. For each scan participants were instructed to avoid alcohol or caffeine ingestion (or any other stimulants or depressants) for at least 12 h prior to testing. Additionally, participants attended MRI testing euhydrated, defined as a urine specific gravity <1.025, which was confirmed prior to imaging using a urine refractometer (Kavouras, 2002). Both MRI scans were performed at the same time of day (±2 h) and participants laid supine for 20 min prior to imaging to minimize the influence of fluid shifts (Berg et al., 1993). An open bore MRI system (Magnetom Aera T-1.5, Siemens, United States) was used to capture the whole thigh from 20 mm superior to the greater trochanter of the femur to 20 mm distal to the proximal aspect of the tibia. Progressive axial scans were performed in 12-mm continuous slices with 10-ms echo time, 525-ms repetition time, 320 × 320 matrix, and 3.2-mm voxel size (Figure 1). Slice locations were normalized to thigh length, which was defined as the distance from the most superior aspect of the femoral head to the most inferior aspect of the femur.

Afterwards a researcher who was blinded to both participants and conditions measured the cross-sectional areas of each of the four QF components (rectus femoris, vastus lateralis, vastus medialis, and vastus intermedius) along the length of the thigh. The measurements used for analysis were taken between 30% (proximal) and 70% (distal) of thigh length with these anatomical locations interpolated between the two closest scan locations using a DICOM analyzer (OsiriX DICOM Viewer, Pixmeo, Switzerland). These end points were selected as the muscle rapidly tapers around these locations and measurements beyond these cut points resulted in reduced measurement reliability. Volumes of each QF component were calculated and then summed to determine Vol_QF_. CoM_QF_ was calculated by integrating muscle volumes between successive scans using a cubic spline method with locations reported relative to thigh length from the most superior aspect of the femoral head. *I*_QF_ was calculated using the equation:

$$I_{QF}=\left({Vol}_{QF}∙m_{k}\right)\;{\left({CoM}_{QF}\right)}^{2}$$

where Vol_QF_ is QF volume, m_k_ is a mass constant of 1.1112 g per cm^3^, and CoM_QF_ is the location of the CoM_QF_ relative to the proximal thigh (Norton et al., 2002; Winter 2009).

### Strength testing

Prior to strength testing, all participants completed a standardized warm-up consisting of 2.5 min of low-intensity (0.5 kp, 60 rpm) cycling with terminal knee extension between 5–15 of flexion (measured by goniometer) followed by eight repetitions of bodyweight squats and eight repetitions on each leg of leg swings, knee hugs, and quadriceps pulls. The participants then performed a single set of six repetitions at 50% their estimated one-repetition maximum load (1-RM).

After warm-up, participants performed strength tests for both unilateral knee extensions and squats on their assigned limbs in the assigned order. For each test, 1-RM was estimated from a repetitions-to-failure test in which the participants performed as many repetitions as possible at an assigned load. The loads selected for each test ensured that participants could perform at least four but no more than 12 repetitions. Only repetitions performed with full range of motion and with proper form were considered successful for the repetition maximum test. From this test, 1-RM was calculated using a previously published repetition-load table (Haff et al., 2016). To compare the relative effort during strength tests between conditions and over time, participants rated their perceived exertion using the Borg CR-10 scale after each test.

The OKC strength test and exercise training were performed on a standard plate-loaded knee extension machine (Model 2COO41BM CC-4, Valor Fitness, United States). On the machine participants were positioned with the femoral lateral epicondyles in line with the axis of rotation and with a starting knee angle of 90 flexion. To ensure a complete range of motion, a low-mass high elasticity string was used as a reference marker and positioned at terminal knee extension; only repetitions in which participants contacted the string were considered successful. Machine settings and reference marker positions were recorded for each participant and for during all subsequent training and testing sessions.

The CKC strength test and exercise training were performed on a Smith Machine (Solid Body PSM144X, Powerline Fitness, United States). Each participant placed their heel directly under their hip and in line with the bar before squatting to a knee angle of 90 flexion. This depth was selected to match the range of motion between the knee extension and squat exercises. Once correct depth was determined, a lightweight high-elasticity string was placed at the bar depth attained at this position and only repetitions in which the bar contacted the string were recorded. Foot position and bar depth were recorded for each participant and repeated for all subsequent training and testing sessions. To ensure both the knee extension and squat tests were performed in line with sagittal plane motion, participants were required to keep their toes pointed forward and their knee in line with the hip and toe throughout each repetition.

### Training program

All participants concurrently completed the resistance training in which they performed both unilateral knee extensions (OKC) and squats (CKC) using contralateral legs. During the 8-week training program, participants trained three times per week on non-consecutive days for a total of 24 training sessions. Participants who missed more than three training sessions (87.5% of sessions) for any reason were excluded from the study, resulting in two participants of the initial 14 recruited being removed from the study for compliance reasons. Throughout the program, loading intensity (65%–85% of 1-RM), repetitions per set (6–12 repetitions), number of sets (three to four sets), and inter-set recovery periods (90–120 s) were manipulated with the goal of facilitating hypertrophy (see Table 1).

**TABLE 1:** Resistance training program details.

Week	Sets	Reps	Load (%)	Rest (s)
1	3	12	65	90
2	3	10	75	90
3	3	8	80	90
4	4	6	85	120
5	4	6	85	120
6	3	12	67	90
7	3	10	75	90
8	3	8	80	90

Program details for both open and closed kinetic chain interventions. Loads are given as a percentage of one repetition maximum but also progressed using the 2-for-2, rule.

Initial training loads were prescribed as a percentage of estimated 1-RM determined from their strength testing results. However, the estimated 1-RM and the training loads calculated using 1-RM load were increased as training progressed in accordance with the 2-for-2 rule, which allows for a 5% increase in estimated 1-RM after two training sessions in which the participant felt that they could have completed two additional repetitions beyond their predicted repetition maximum for that load (Haff et al., 2016). The utilization of the 2-for-2 rule allowed for absolute training loads to be progressed in a conservative manner, which accounts for increases in strength gains but removes the need for repeated 1-RM testing. This also allows the relative intensity of each load to remain appropriate to the training goals of an individual training session.

### NIRS measurements

During the second week of training microvascular changes in hemoglobin concentration and tissue oxygenation were measured while participants completed one of their assigned workouts for both conditions. A multiple distance, frequency domain NIRS system (OxiPlexTS, ISS, Boston, United States) was used. This NIRS system utilizes a unique method of separating signal scattering and absorption (determined through measured scattering coefficients and application of the theory of photon migration) that allows for direct absolute measurements of oxygenated hemoglobin (HbO_2_), deoxygenated hemoglobin (HHb) and oxygen saturation (StO_2_) (Franceschini et al., 1998; Hueber et al., 1999).

During the session, participants completed their normal cycling warm-up before being outfitted with two rigid muscle NIRS probes placed over their proximal and distal vastus lateralis (30% and 70% thigh length). Each probe utilized 16 laser diode emitters (690 and 830 nm wavelengths) and eight detectors with emitter-detector distances were 2.0, 2.5, 3.0 and 3.5 cm. Collected NIRS signals at an analog-digital conversion rate of 20 Hz. The probes were initially calibrated following manufacturer instructions then placed on the muscle belly using medical adhesive spray and Velcro straps. The NIRS probes were then covered with a lightweight fabric designed by the NIRS manufacturer to prevent the infiltration of ambient light during the monitoring.

Subsequently, participants assumed a resting position specific to the exercise condition: the resting position for CKC was standing unsupported with load equally distributed between feet, while the resting position for OKC was seated in the leg extension machine with their leg resting against the pad of the machine. In this position participants rested for 5 min before 3 min of baseline NIRS measurements were obtained. Participants then started the bout of exercise, which consisted of three sets of 10 repetitions with a load of 75% of 1-RM with 90 s of passive rest between sets. Throughout the exercise and during five consecutive minutes of passive recovery NIRS data were continually collected. Then, participants repeated the testing with the other leg and exercise condition. Average HbO_2_, HHb, and StO_2_ during the last set of exercise were used for statistical analyses.

### Statistical analysis

Measurement reliability for the primary dependent variables (Vol_QF_, CoM_QF_ and *I*_QF_) were determined by calculating intraclass correlation coefficients for absolute agreement between pre-intervention measurements on CKC to OKC limbs. To determine whether any differences in Vol_QF_, CoM_QF_ or *I*_QF_ existed between limbs (CKC vs OKC) before training, pre-training values were compared between conditions using paired t-tests.

A 2 × 2 (condition × time) repeated measures MANOVA with Bonferroni *post hoc* correction was used to assess interactions between training condition (OKC vs CKC) and time (pre-to post-training) on Vol_QF_, CoM_QF_ and *I*_QF_ and to interrogate changes over time within each condition, with an additional 2 × 2 repeated measures MANOVA performed with Bonferroni *post hoc* corrections to compare differences between conditions in rating of perceived exertion (Borg CR-10) during strength tests and within each condition over time for estimated 1-RM strength. A separate 2 × 2 (condition × region) repeated measures MANOVA with Bonferroni *post hoc* correction was used to compare NIRS outcome measures between conditions (OKC vs CKC), regions (proximal and distal thigh), and interaction between conditions and regions. Results from both MANOVAs were checked for sphericity using a Greenhouse-Geisser test with Huyn-Feldt corrected results reported when data violated sphericity.

To determine the potential value of comparing regional (proximal vs distal) NIRS measures (HbO_2_, HHb and StO_2_) between exercise conditions (OKC vs CKC) in a single session to “predict” changes in CoM_QF_ and *I*_QF_ in response to 8 weeks of training, two linear stepwise regressions were performed. The outcome of the regressions were the ratio of changes in CoM_QF_ and *I*_QF_ between OKC and CKC (Δ*I*_QF.OKC_: Δ*I*_QF.CKC_ and ΔCoM_QF.OKC_: ΔCoM_QF.CKC_). The inputted predictors of the regressions included the ratio of OKC to CKC differences in regional (proximal—distal) NIRS outcomes [e.g (HHb_OKC.Prox_ - HHb_OKC.Distal_): (HHb_CKC.Prox_—HHb_CKC.Distal_)].

Data are presented as means, standard deviations and percentage changes from baseline unless otherwise noted. Effect sizes were determined using Cohen’s D. Statistical analyses were completed using SPSS version 24.0 (IBM, New York United States). Statistical significance was accepted at an α level of P ≤ 0.05. To estimate the necessary sample size for this study, an *a priori* power analysis was performed using G*Power version 3.1.9.6. The MANOVA with repeated measures for within-between interaction test was selected. The input values were: two groups, two repeated measurements, adequate statistical power of 0.85, and α level = 0.05, which are typical in research of this type. The estimated effect size f (V) = 1.0 was derived from the previously mentioned pilot data comparing regional activation between OKC and CKC and is consistent with previous studies (Franchi et al., 2014).

## Results

Twelve participants (7 men: age: 22.3 ± 4.4 years, height: 171.0 ± 7.6 cm, body mass: 66.5 ± 9.5 kg, % body fat: 14.2 ± 4.6, and five women, age: 19.8 ± 0.8 years, height: 163.8 ± 4.3 cm, body mass: 58.6 ± 6.3 kg, % body fat: 24.4% ± 3.1%) completed the study. Of the 24 total training sessions, participants completed 23.7 ± 0.5 sessions. Before training commencement, no differences were observed between conditions in thigh length (CKC: 46.7 ± 2.4 cm, OKC: 46.1 ± 2.5 cm), Vol_QF_ (CKC: 1042 ± 284 cm^3^, OKC: 1085 ± 394, *p* = 0.350), CoM_QF_ (CKC: 20.0 ± 2.1 cm, OKC: 20.0 ± 1.9 cm, *p* = 0.940) or *I*_QF_ (CKC: 0.0879 ± 0.0337 kg m^2^, OKC: 0.0896 ± 0.0356 kg m^2^: *p* = 0.490). Reliability assessment of the primary dependent variables revealed that Vol_QF_ (ICC = 0.950, *p* < 0.001), CoM_QF_ (ICC = 0.982, *p* < 0.001) and *I*_QF_ (ICC = 0.985, *p* < 0.001) could be reliably obtained.

Results of the strength tests indicate that estimated 1-RM increased for both CKC (Pre: 37.9 ± 13.9 kg, Post: 70.27 ± 23.0 kg; *p* < 0.001) and OKC (Pre: 34.5 ± 11.5 kg, Post: 52.1 ± 13.7 kg, *p* < 0.001) after training. However, as the strength test exercises differed between legs, no comparisons between conditions were made. Rating of perceived exertion during strength tests did not differ between CKC (pre: 6.6 ± 1.7, post: 7.4 ± 1.4) and OKC (pre: 7.0 ± 1.7, post: 7.1 ± 1.3; *p* = 0.795) or over time (*p* = 0.312).

**FIGURE 2:**
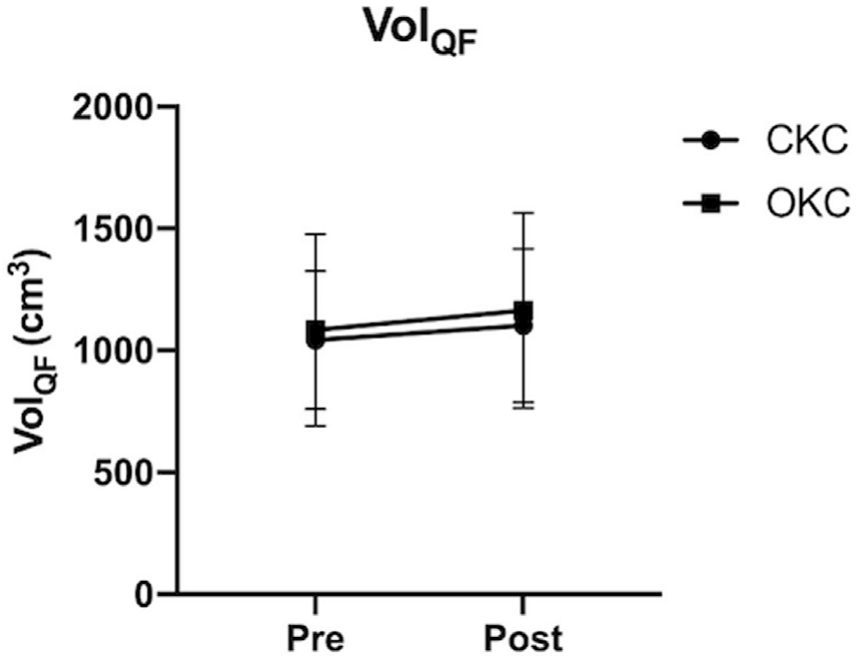
Group average (*n* = 12) changes in quadriceps femoris volume (VOL_QF_) in response to 8 weeks of open (OKC) and closed-kinetic chain (CKC) exercise. Mean and standard deviation are reported. *significant differences (*p* ≤ 0.05) between interventions over time (interaction effect). ^#^significant difference within conditions over time.

**FIGURE 3:**
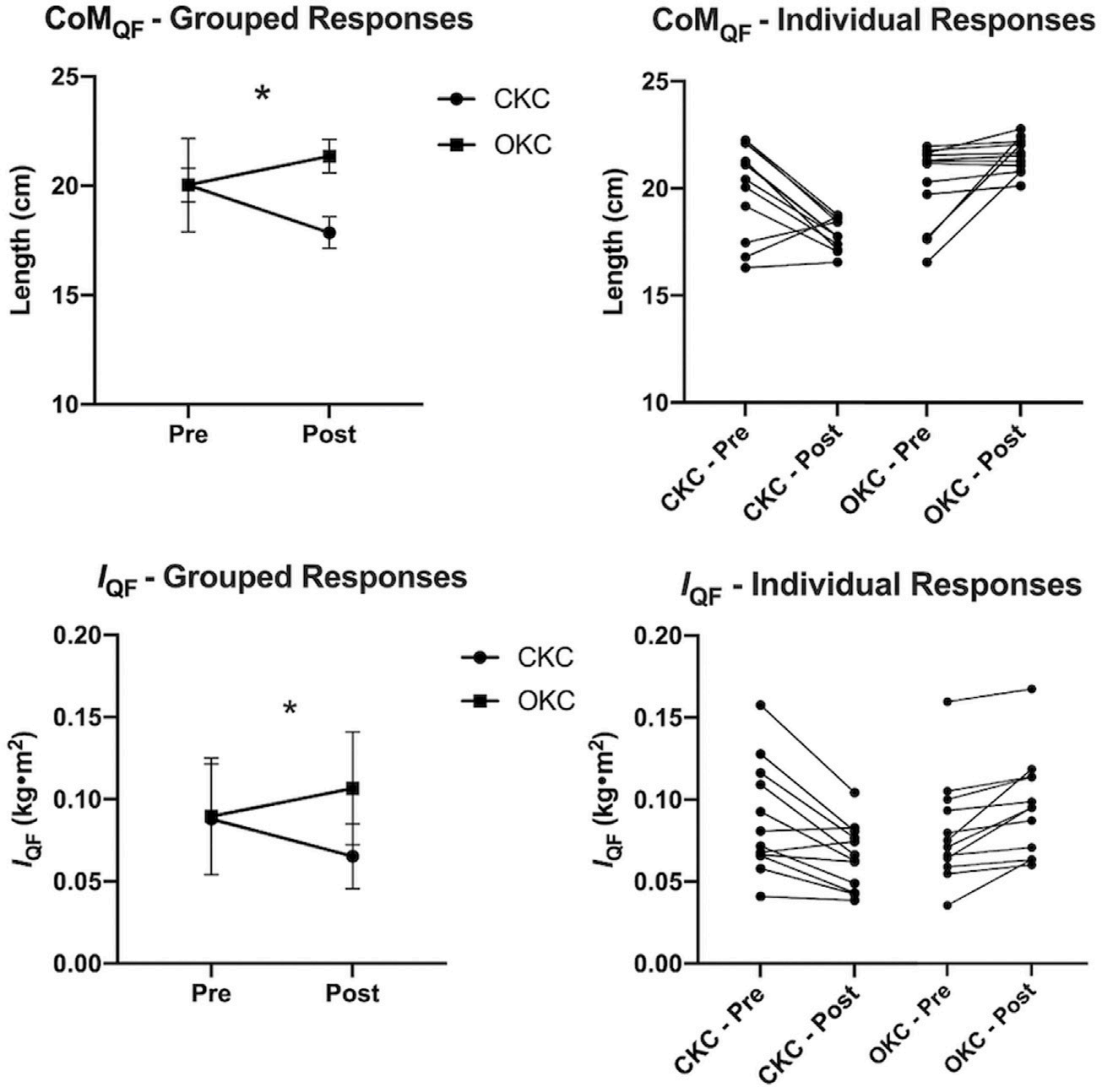
Grouped (left) and individual (right) changes in quadriceps femoris center of mass location (CoM_QF_: top) and moment of inertia (I_QF_: bottom) relative to the hip, in response to 8 weeks of open (OKC) and closed-kinetic chain (CKC) exercise. Mean and standard deviation are reported with significant differences (*p* ≤ 0.05) between interventions over time (interaction effect) are represented by * (*n* = 12).

Grouped changes in Vol_QF_ and both group and individual changes in CoM_QF_ and *I*_QF_ over time are shown in Figures 2, 3, respectively. Additionally, changes in volume of each QF muscular component are provided in Supplementary File S2. While no differences between CKC (Δ5.8% ± 5.4%, d = 0.20) and OKC (Δ8.4% ± 9.5%, d = 0.20) were observed over time for Vol_QF_ (interaction effect; *p* = 0.477), significant differences between conditions over time were observed for changes in CoM_QF_ (*p* < 0.001) and *I*_QF_ (*p* < 0.001). For the OKC condition a significant distal shift in CoM_QF_ (Δ2.4 ± 0.4 cm, *p* < 0.001, d = 1.72) and an increase in *I*_QF_ (Δ0.017 ± 0.014 kg m^2^, *p* < 0.001, d = 0.49) occurred over time, however these values did not significantly change in the CKC condition (CoM_QF_: Δ-2.2 ± 2.0 cm, *I*_QF_: Δ-0.022 ± 0.020 kg m^2^, *p* > 0.05) over time.

**FIGURE 4:**
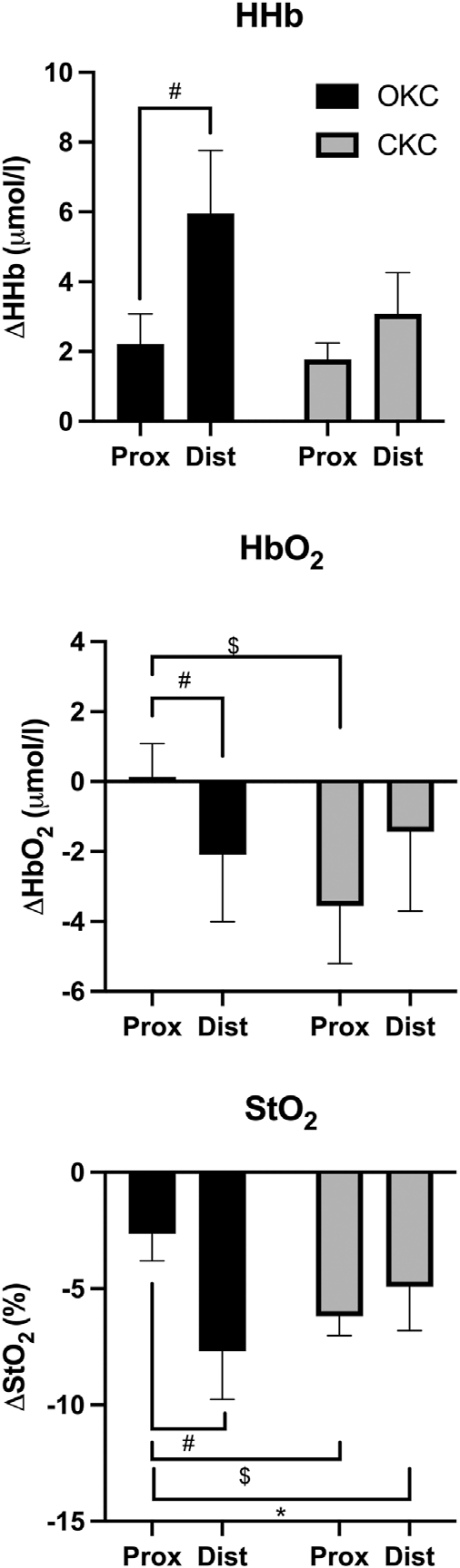
Group average (*n* = 12) changes in deoxygenated (HHb) and oxygenated (HbO_2_) hemoglobin concentration and in tissue oxygenation saturation (StO_2_), during open-kinetic chain (OKC) and closed-kinetic chain (CKC) training sessions. * Mean and standard measurement error are reported with significant differences (*p* ≤ 0.05) between interventions over time (*), between regions for a given exercise (#) and between exercises within a region ($) depicted.

Comparisons of NIRS data between locations and exercise conditions are shown in Figure 4. During the final set of exercise, a significant interaction effect was observed for StO_2_ (F = 5.823, *p* = 0.034) but not for HbO_2_ (*p* = 0.225) or HHb (*p* = 0.225). In the proximal region, greater reductions in HbO_2_ (*p* = 0.018, d = 0.79) and StO_2_ (*p* = 0.007, d = 1.06) were observed in CKC than OKC. Additionally, statistically greater decreases in HbO_2_ (*p* = 0.008, d = 0.42) and StO_2_ (*p* = 0.011, d = 1.06) and increase in HHb (*p* = 0.032, d = 0.76) were observed in OKC distally than proximally.

**FIGURE 5:**
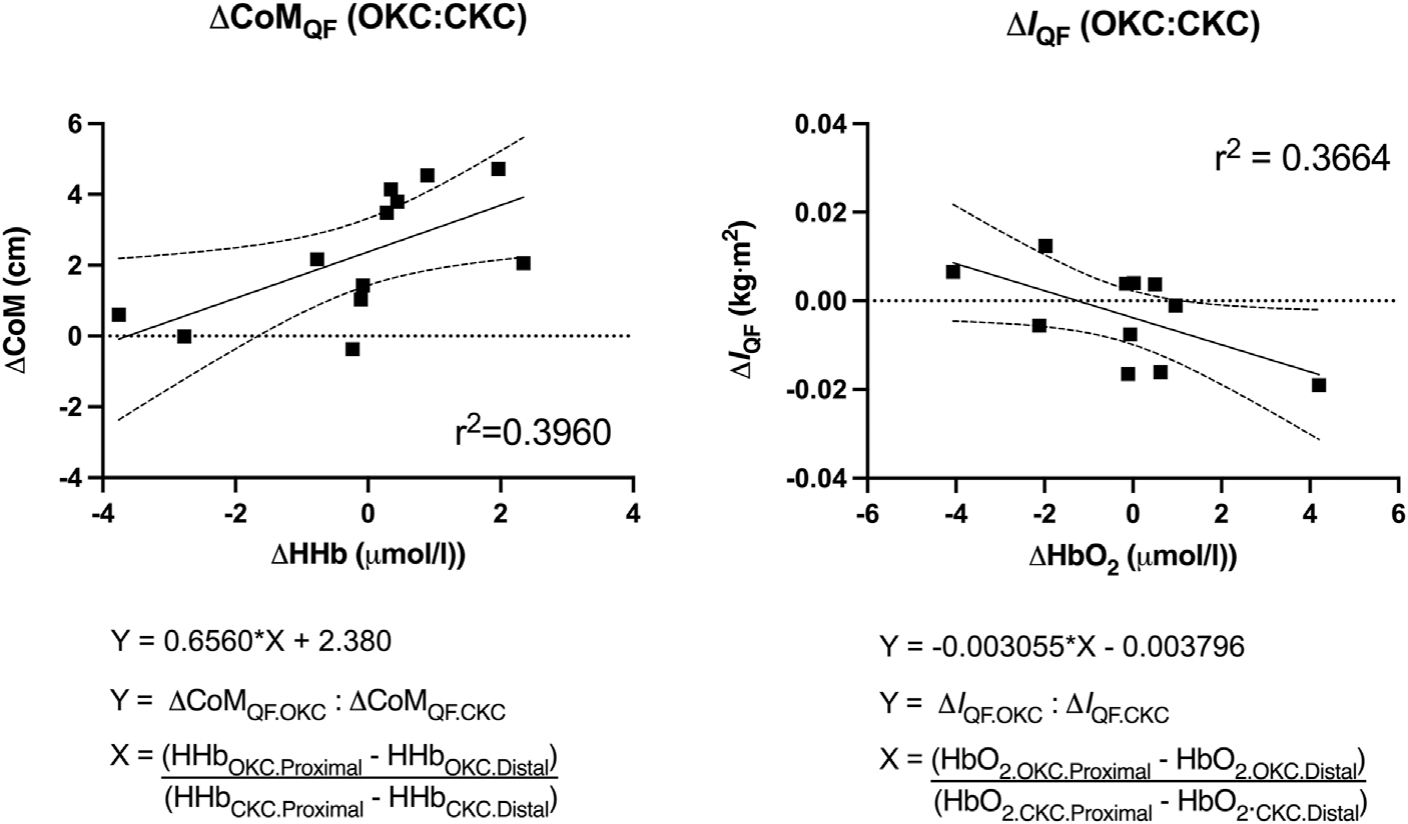
Predictive value of regional differences in near-infrared spectroscopy measures [deoxygenated (HHb) and oxygenated (HbO_2_)] within a single bout of open (OKC) and closed-kinetic chain exercise (CKC) exercise in vastus lateralis on changes in quadriceps femoris center of mass (CoM_QF_) and moment of inertia (I_QF_) after 8-week of training (*p* < 0.05).

Results from the regression analyses are shown in Figure 5. The differing response to OKC vs CKC training in CoM_QF_ was best predicted by regional differences in HHb with 36.6% of the observed variance explained (*p* = 0.049), while the training response to *I*_QF_ was best predicted by regional differences in HbO_2_ with 39.6% of the observed variance explained (*p* = 0.028). However, the inclusion of additional NIRS variables provided no enhanced predictive strength.

## Discussion

The main finding of the present study was that OKC (leg extension) and CKC (squat lift) exercises, which both target the knee extensors, induced different patterns of regional hypertrophy that altered muscle shape sufficiently to influence both center of mass location (CoM_QF_) and moment of inertia (*I*_QF_) about the hip. Specifically, CKC induced a more proximal pattern of hypertrophy compared to OKC, resulting in a concomitant proximal shift in CoM_QF_ and decrease in *I*_QF_, despite a similar overall increase in VOL_QF_ being evoked in both conditions. Given *I*_QF_ is inversely related to running economy and performance, these results suggests that CKC produces a more favorable pattern of hypertrophy for running than OKC.

A secondary finding of the present study was that the observed effects of exercise selection on CoM_QF_ and *I*_QF_ also occurred in a manner that was partially predicted by pre-training NIRS-based assessments of muscle microvascular blood flow and oxygenation. These results highlight the potential use of NIRS as a tool for predicting patterns of hypertrophy between different exercises and conditions.

While previous studies have reported differential patterns of regional hypertrophy between exercises with different movement patterns (Wakahara et al., 2012; Wakahara et al., 2013; Nunes et al., 2020), loads, movement velocities (Earp et al., 2015) and contraction modes (Franchi et al., 2014), this is the first study to show that such changes can meaningfully alter both CoM_QF_ and *I*_QF_. Additionally, this is the first study to demonstrate that the moment of inertia of a muscle may remain unaltered despite an increase in muscle volume and mass due to a redistribution of mass along the length of the muscle. As the quadriceps femoris is a primary agonist during locomotion, many activities of daily living and sporting tasks and has been shown to undergo significant hypertrophy in response to resistance training, understanding how different versions of exercise affect muscle shape and mass distribution may have significant practical implications for athletic performance (Delecluse, 1997; Franchi et al., 2014; Earp et al., 2015).

It is well established that the mass distribution of a limb (relative to its axis of rotation) strongly impacts joint kinetics, with more proximal distribution reducing joint loads and energy demands when moving with a fixed velocity and increasing angular acceleration when moving with given moment of force (Myers and Steudel, 1985; Hale, 1990; Winter 2009). As such, utilizing exercise programs that preferentially induce proximal hypertrophy may provide a direct biomechanical benefit to limb motion (Handsfield et al., 2017). However, it is important to note that the pattern of hypertrophy deemed most suitable will depend on the movement being performed because it will differ based on the location of the rotational axis. Using the example of human running, a proximal pattern of hypertrophy might be considered preferential during the swing phase as it reduces moment of inertia about the hip (*I*_QF_) but a distal pattern may be beneficial during the stance phase (as the thigh rotates over the foot); distal hypertrophy may also be of benefit in other tasks, e.g., when rising from a chair. However, as the thigh contributes to a larger proportion of the total moment of inertia during the swing phase than the stance phase, a proximal pattern of hypertrophy should ultimately be beneficial for running (Handsfield et al., 2017). This hypothesis has been supported by previous studies reporting that faster runners possess greater proximal and lesser distal muscle mass than slower runners at both the 100 m and marathon distances (Abe et al., 2000; Kumagai et al., 2000; Miller et al., 2021) as well as compared to non-sprinters (Handsfield et al., 2017). Additionally, results are equivocal as to whether QF mid-thigh cross-sectional area and muscle volume are related to running performance, with several studies failing to find a relationship (Sugisaki et al., 2011; Handsfield et al., 2017; Sugisaki et al., 2018) but one finding such a relationship (Miller et al., 2021).

While the mechanisms driving region-specific hypertrophy are yet to be explicitly determined, several potential factors have been identified, including proximo-distal variation in muscle architecture (fascicle angle, fascicle length and cross-sectional area), fiber type distribution, and selective muscle/regional activation (Wakahara et al., 2012; Wakahara et al., 2013; Franchi et al., 2014; Earp et al., 2016; Wakahara et al., 2017). The variations in all of these components have been previously hypothesized to allow for specific portions of a muscle to specialize in specific exercise conditions (Wakahara et al., 2017). Such specialization is particularly important as the present study compared two exercises that present with distinct movement kinetics and muscle loading patterns, even when similar external loads (% 1-RM) are lifted for the same repetitions, at similar speeds (Wallace et al., 2018). For instance, because CKC presents with greater loading during the portion of the exercise while the muscle is operating at long muscle lengths when compared to OKC, regions of the muscle that are specialized for these conditions, such as those with longer fascicle lengths will contribute greater to CKC than OKC (Hinks et al., 2022). Regarding intramuscular variations in fiber type, it is unlikely this factor contributed to the observed results as the relative intensity, training duration, and speed of movement were similar between conditions and there are no reports of significant functional compartmentalization within the QF that would indicate that some regions would be of a more prominent fiber type (Burkholder et al., 1994). In contrast, selective activation of specific regions of a muscle cannot be discounted. While previous studies have found that muscle activity measured using EMG was unrelated to patterns of hypertrophy (Miyamoto et al., 2013; Earp et al., 2016), others have found that patterns of hypertrophy can be predicted using T_2_-weighted MRI (Wakahara et al., 2012; Wakahara et al., 2013; Wakahara et al., 2017). Unlike EMG, which measures the propagation of electrical signals throughout the muscle during exercise, T_2_-weighted MRI measures changes in intramuscular fluid accumulation after exercise. Given these differences, it is likely that physiological stress, specifically changes in muscle oxygenation, blood flow, and metabolism, rather than neurological activity itself, drives intramuscular hypertrophy (Miyamoto et al., 2013).

In the present study, NIRS was used to assess changes muscle microvascular blood flow and oxygenation between exercises. Similar to the T_2_-weighted MRI measurements, NIRS reflects physiological stress independent of neurological activity by directly measuring changes in muscle oxygenation (Chavhan et al., 2009). However, NIRS provides several logistical advantages to MRI such as the ability to measure changes in muscle oxygenation saturation during large body movements and a reduced cost of measurement. In the present study, two NIRS sensors were placed in the proximal and distal portions of the vastus lateralis component of the quadriceps femoris. As each sensor obtained measurements over a 3.5 cm region of interest, approximately 20% of the length of the quadriceps femoris muscle belly measured by MRI images was covered by the two sensors. However, despite NIRS measurements being taken from only a portion of a single component of the total quadriceps femoris, these signals were able to effectively predict differences in the changes in CoM_QF_ (39.6% of variance) and *I*_QF_ (36.6% of variance) between the exercises; this is despite the measurements reflecting the summative changes to quadriceps femoris, which is a complex three-dimensional structure. Our finding that NIRS-derived vastus lateralis measurements (specifically HbO_2_ and HHb) were associated with regional hypertrophic magnitudes caused by different training stimuli presents NIRS as a potential tool for predicting patterns of hypertrophy between other exercises and conditions. These results demonstrated that individuals who experienced differing changes in either CoM_QF_ or *I*_QF_ after completing both interventions also presented with differences in NIRS measurements taken from a single training session early in their training intervention. For instance, given the positive relationship depicted in Figure 5, it was observed that individuals who experienced a more distal shift in CoM_QF_ in their OKC limb compared to their CKC limb (larger value on *Y*-axis) were likely to also present with a greater discrepancy between limbs in HHb accumulation in the 5 min after the exercise driven either by greater distal HHb after OKC or greater proximal HHb after CKC (larger *X*-Axis).

It is important to note that CoM_QF_ and *I*_QF_ changes were computed using only QF muscle mass data and do not include other muscles, bone or adipose. While changes in whole thigh center of mass and moment of inertia about the hip are of great practical interest, such comparisons were not appropriate in the present study as both the knee extensors and hip extensors are utilized during CKC and only the knee extensors are agonists during OKC; the comparison was therefore limited to the common quadriceps muscle group. Another important note is that the role and function of the rectus femoris differed between exercise conditions as the rectus femoris is biarticular and CKC is a compound movement where this muscle would contract concurrently (lengthening at one joint while shortening at another) and thus serve more as a stabilizer than a work producing agonist (Escamilla et al., 1998). As such differences in rectus femoris activity may have contributed to the observed differences in CoM_QF_ and *I*_QF_ between exercises. However, surprisingly we observed similar increases in rectus femoris muscle volume between exercise conditions (see Supplementary Table S1), which contrasts previous observations that CKC elicits minimal hypertrophy of the rectus femoris (Earp et al., 2015). One possible explanation for this discrepancy is that CKC in the present study was performed unilaterally and thus had greater stability demands than the bilateral motions used in previous studies.

The present study utilized a within subject design as a means of strengthening statistical power while also accounting for individual responsiveness to training. However, this design comes with several limitations. Firstly, interpretation of the differences between limbs assumes both limbs share similar qualities such as baseline strength, muscle structure and fiber composition. Secondly, the lack of a control group prevents from determining the individual training effects of either intervention. Finally, as both training interventions were performed concurrently we cannot exclude a cross-over effect is present in both training groups. To this final point, despite crossover effects being credited to neurological cross-education that occurs independent of hypertrophy (Hendy and Lamon, 2017) such an effect may have affected our results. Specifically, because bilateral training results in significantly greater anabolic response than unilateral training (Migiano et al., 2010), and both trainings were performed in immediate succession, received an atypical hypertrophy response may have occurred. Another limitation of the present study was that NIRS measurements of the vastus lateralis were used as the representative measure for the entire quadriceps femoris. As such it is likely that the observed predictive value of NIRS was weakened compared to other methods which could assess all four components of the quadriceps femoris. A final delimitation of the present study was that the participants were not habitually resistance trained and not concurrently taking part in other forms of training (e.g., running); therefore, additional research is necessary to determine whether similar adaptive patterns exist in individuals performing other concurrent training modalities.

In summary, CKC knee extensor training resulted in a more proximal QF hypertrophic pattern than OKC squat lift training, which resulted in the center of mass (CoM_QF_) being located more proximally and a smaller moment of inertia (*I*_QF_) after training. These differences may have important practical application to running performance given the known relationship between *I*_QF_ and running performance and economy. Specifically, strengthening exercises that target more proximal patterns of hypertrophy, such as CKC, may be preferential for running athletes. However, additional research is needed to determine the potential clinical significance of these findings by directly measuring the effects of differing patterns of hypertrophy resulting on running performance. The present study also observed that individual changes in CoM_QF_ and *I*_QF_ between exercises could be partially predicted by pre-training NIRS measurements of muscle microvascular blood flow and oxygenation in the proximal and distal portions of vastus lateralis. These results highlight the potential of NIRS as a tool for predicting patterns of hypertrophy between different exercises and conditions.

## Data Availability

The original contributions presented in the study are included in the article/Supplementary Material, further inquiries can be directed to the corresponding authors.
