# Supplementary material for: Differing hypertrophy patterns from open and closed kinetic chain training affect quadriceps femoris center of mass and moment of inertia
Source: Front Physiol. 2023 Mar 14;14:1074705. doi: 10.3389/fphys.2023.1074705 (PMC10043166; doi:10.3389/fphys.2023.1074705)
Supplement: Supplementary file 1 [file Table1.DOCX]

**Supplementary File 2**

Muscle volumes measured before (Pre) and after (Post) training in CKC and OKC, with change scores (%) and p values for the time × muscle interaction effect.

|  | CKC | | | OKC | | | Interaction |
| --- | --- | --- | --- | --- | --- | --- | --- |
| Muscle | Pre (cm^3^) | Post (cm^3^) | Change (%) | Pre (cm^3^) | Post (cm^3^) | Change (%) | P value |
| RF | 231.4 ± 62.3 | 268.7 ± 66.9 | 16.7 ± 7.5* | 239.1 ± 71.8 | 262.4 ± 76.3 | 10.7 ± 14.5* | 0.376 |
| VL | 555.2 ± 163.6 | 597.5 ± 141.4 | 11.7 ± 25.5 | 564.9 ± 177.9 | 616.6 ± 118.3 | 14.5 ± 21.7 | 0.021* |
| VM | 404.1 ± 160.3 | 457.3 ± 156.2 | 16.5 ± 18.2 | 392.9 ± 119.7 | 454.7 ± 125.0 | 17.6 ± 17.0 | 0.895 |
| VI | 526.0 ± 168.8 | 548.5 ± 151.5 | 6.9 ± 17.0 | 504.2 ± 152.8 | 558.7 ± 122.9 | 15.8 ± 23.8 | 0.124 |
| Total | 1717.2 ± 531.6 | 1871.3 ± 473.0 | 11.2 ± 15.4 | 1702.2 ± 492.2 | 1892.4 ± 409.5 | 14.0 ± 14.9 | 0.477 |

Data are mean ± SD. CKC: Closed kinetic chain, OKC: Open kinetic chain, RF: Rectus femoris, VL: Vastus lateralis, VM: vastus medialis, VI: vastus intermedius.
